# An explainable hybrid feature aggregation network with residual inception positional encoding attention and EfficientNet for cassava leaf disease classification

**DOI:** 10.1038/s41598-025-95985-w

**Published:** 2025-04-06

**Authors:** M. Sundara Srivathsan, S. Alden Jenish, K. Arvindhan, R. Karthik

**Affiliations:** 1 School of Electronics Engineering, Vellore Institute of Technology, Chennai, India; 2 Centre for Cyber Physical Systems, Vellore Institute of Technology, Chennai, India

**Keywords:** Cassava leaf disease, Explainable AI, Deep learning, Convolutional neural network, Image classification, Engineering, Computational science, Computer science

## Abstract

Cassava is a tuberous edible plant native to the American tropics and is essential for its versatile applications including cassava flour, bread, tapioca, and laundry starch. Cassava leaf diseases reduce crop yields, elevate production costs, and disrupt market stability. This places significant burdens on farmers and economies while highlighting the need for effective management strategies. Traditional methods of manual disease diagnosis are costly, labor-intensive, and time-consuming. This research aims to address the challenge of accurate disease classification by overcoming the limitations of existing methods, which encounter difficulties with the complexity and variability of leaf disease symptoms. To the best of our knowledge, this is the first study to propose a novel dual-track feature aggregation architecture that integrates the Residual Inception Positional Encoding Attention (RIPEA) Network with EfficientNet for the classification of cassava leaf diseases. The proposed model employs a dual-track feature aggregation architecture which integrates the RIPEA Network with EfficientNet. The RIPEA track extracts significant features by leveraging residual connections for preserving gradients and uses multi-scale feature fusion for combining fine-grained details with broader patterns. It also incorporates Coordinate and Mixed Attention mechanisms which focus on cross-channel and long-range dependencies. The extracted features from both tracks are aggregated for classification. Furthermore, it incorporates an image augmentation method and a cosine decay learning rate schedule to improve model training. This improves the ability of the model to accurately differentiate between Cassava Bacterial Blight (CBB), Brown Streak Disease (CBSD), Green Mottle (CGM), Mosaic Disease (CMD), and healthy leaves, addressing both local textures and global structures. Additionally, to enhance the interpretability of the model, we apply Grad-CAM to provide visual explanations for the model’s decision-making process, helping to understand which regions of the leaf images contribute to the classification results. The proposed network achieved a classification accuracy of 93.06%.

## Introduction

Cassava is a woody plant commonly found in tropical regions of Africa, Asia, and Latin America. Its adaptability to harvesting times, low water requirements, and capacity to survive in marginal soils have made it popular^1^. The crop offers important qualities that millions of Africans benefit from, such as improved nutritional value and increased resistance to drought and pests, ensuring a more reliable food supply in challenging climates. Cassava plants have high rates of carbon dioxide assimilation to sugar and a high temperature optimum for photosynthesis. Yields of cassava cultivated with conventional tropical techniques range from 5 to 20 tons per hectare, and with the right cultivation techniques, they can reach above 60 tons per hectare^2^. Reports from the International Mining and Resources Conference state that the size of the world market for cassava processing reached 319.9 million tons in 2023. The market is projected to grow at a Compound Annual Growth Rate (CAGR) of 1.4% from 2024 to 2032, reaching 369.7 million tons by the end of the period^3^. Biologic factors, particularly Cassava Mosaic Disease (CMD) produced by Cassava Mosaic Gemini viruses (CMGs) and CBSD induced by Cassava Brown Streak Viruses (CBSVs), have seriously threatened the quality, market yield, productivity, and value of this essential crop. CMD results in leaf deformation, mottling, and yellow mosaic coloring on the leaves, as well as a reduction in leaf and plant size^4^. On the other hand, two plant RNA viruses that can arise together or independently produce necrotic rot of the roots in CBSD^5^. Xanthomonas axonopodis pv. is the cause of CBB. Manihotis, which is also the sixth most dangerous pathogenic bacterium, can result in yield reductions that range from 12 to 95%^6,7^. Pests known as cassava green mottles feed mostly on young leaves, stunting their growth and decreasing their ability to photosynthesize, keeping them tiny, pale, and mottled^5,8^.

To prevent the spread of these diseases, various control strategies including cultural practices, biological interventions, and chemical treatments have been rigorously implemented. However, conventional methods of manual disease diagnosis with the help of agricultural specialists are time-consuming and labor-intensive. Consequently, there is a need to adopt the latest technologies to overcome these limitations^9^. The implementation of automated diagnostic systems developed using Computer-Aided Diagnosis (CAD) can effectively address these constraints. These systems employ digital image processing and computer vision techniques, delivering a cost-effective, efficient, and accurate approach to disease diagnosis^10^. This technique has gained considerable attention due to recent advancements in Machine Learning (ML) and Deep Learning (DL), which have significantly enhanced the efficiency of image classification^11,12^. Identification of the diseases at an early stage prevents their spread and mitigates the chances for total crop failure, reducing significant economic loss. Although ML approaches have been employed to classify cassava diseases, researchers have transitioned to DL techniques in order to achieve superior results. This is because, in comparison to DL approaches, ML algorithms are limited by small dataset sizes and need more data for accurate classification. Although limited dataset size is a challenge for DL, data augmentation techniques can help overcome this problem. Furthermore, deep learning techniques eliminate the need for manual feature extraction by automatically extracting features, unlike traditional machine learning methods. DL techniques are extensively employed today to develop solutions for prediction and classification-related challenges. While each DL model aims to enhance accuracy, they often overlook the computational costs associated with the architecture. With few exceptions, limited research has explored various ML architectures. Additionally, the datasets commonly employed are typically imbalanced, with a greater number of samples belonging to one class compared to others. This study presents an automated system for classifying cassava leaf diseases through a dual-track DL network. The proposed network is trained using image data that includes healthy leaves along with CGM, CBSD, CBB and CMD.

## Related works

Extensive research has been conducted on developing automated systems for detecting diseases in plant leaves across different species^13^. The following section reviews previous studies in cassava leaf disease detection. DL algorithms can process raw input data directly, removing the requirement for manually crafted features. With the aid of graphics processing units and high-performance computation, DL models can now be trained effectively by utilizing parallelism. Recent and ongoing research have focused on developing DL networks for classifying plant leaf diseases^14,15^. Various DL models were used to train leaf image samples for disease identification. Many studies leverage state-of-the-art architectures, including VGG16, ResNet, AlexNet, and GoogleNet, to detect cassava leaf infections. Surya et al. combined Convolutional Neural Network (CNN) and the MobileNet V2 architecture with the ReLU activation function and Softmax Classifier^16^. Calma et al. proposed an image-based system for identifying cassava leaf and stem diseases using MobileNetV3 with dataset augmentation. This system enables the classification of five distinct categories of leaf diseases that affect cassava plants^17^. Pandey et al. employed an Attention Dense Learning (ADL) mechanism that combines mixed sigmoid attention learning with the dense learning process of CNNs to enhance the identification of diseases from in-field RGB images. This approach allows the model to achieve better classification accuracy by distinguishing significant lesion features from the background clutter^18^. Singh et al. leveraged a transfer learning approach where classification of diseases is achieved using a DL model called DenseNet169^19^. Similarly, Emmanuel et al. introduced a model that incorporates a transfer learning technique, utilizing a deep Gaussian CNN. This model was then evaluated against both the squared exponential and rational quadratic kernels for comparison^20^. Riaz et al. leveraged augmentation techniques to increase the number of samples for classification and balance the unequal data distribution for all classes^21^. Furthermore, the EfficientNetB3 model was used for identification and classification. Ahishakiye et al. introduced an innovative approach using spectral data to classify cassava diseases in a three-class diagnostic task. Additionally, they proposed an ensemble model named Generalized Matrix Learning Vector Quantization (GMLVQ), which is derived from Generalized Learning Vector Quantization (GLVQ)^22^.

In recent years, deep residual networks and attention mechanisms have been explored in classifying diseases with high accuracy. Xiao et al. incorporated the Squeeze-and-Excitation Variant Residual Network (SE-VRNet), a lightweight model that integrates a Squeeze-and-Excitation module with an attention mechanism and a residual network. This model, based on an attention mechanism and a residual network, improved the extraction of accurate lesions and regions of interest. It resolved the issue of difficult feature extraction due to the dispersed distribution of leaf diseases^23^. For accurate classification, it is also necessary to create deep neural networks that are tailored to the target domain. Sambasivam et al. tackled class imbalance using methods like class weighting and the Synthetic Minority Oversampling Technique (SMOTE). To boost disease detection accuracy, focal loss was combined with a tailored deep CNN architecture^24^. Oyewola et al. also utilized a custom CNN model featuring residual connections, outperforming standard CNN models in terms of performance, particularly in distinguishing between different cassava diseases^25^. Furthermore, Hassan et al. introduced an altered CNN architecture built on the Inception-V3 model, demonstrating its ability to identify cassava diseases by utilizing advanced feature extraction techniques^26^. In Karthik et al. a deep fusion model combining EfficientNet and a residual channel shuffled attention network was proposed. The model utilized depthwise separable convolution for contextual information extraction and integrated spatial and channel data through the triplet attention module for feature extraction^27^. Patike et al. introduced an approach using depthwise separable convolution layers to improve feature extraction^28^. In another study, Maryum et al. adopted the UNet model to eliminate background noise, subsequently applying a pre-trained EfficientNet-B4 model for classification^29^. These approaches combine segmentation with advanced classification techniques to enhance the accuracy of cassava disease detection. The aforementioned techniques shows that DL models are employed to identify cassava diseases, reflecting current trends and practices in the field. However, this study has pinpointed specific drawbacks such as class imbalance, lower accuracy, prolonged processing times, and inadequate focus on essential leaf characteristics. The subsequent section explores these gaps and outlines the measures implemented to overcome these limitations in the proposed research.

### Research gaps and motivation

The proposed work addresses the following research gaps in the detection of cassava leaf diseases.

1. Existing studies use datasets that exhibit significant class imbalance, which affects the ability of the model to learn critical feature patterns effectively. Many studies are limited in incorporating effective techniques beyond augmentation to address the class imbalance present in the dataset, resulting in biased model performance.
2. Most current research in cassava disease detection relies on traditional CNN models and pre-trained methods. Developing a tailored architecture unique to the specific characteristics of the input data will enhance the generalization capabilities of the model.
3. Existing research treats all channels with equal weight, overlooking their varying importance. They do not accurately assign specific weights to individual feature maps, whether within a single channel or among multiple channels. Furthermore, incorporating contextual information is essential for accurately identifying and understanding the relationships between neighboring pixels.

### Research contributions

The following are the main contributions of the proposed work in the cassava leaf disease classification.

1. To tackle the issue of class imbalance, the proposed approach employs class weights. Class weights assist in mitigating the impact of imbalanced data during training by applying greater penalties for misclassifications of minority classes. This approach encourages the model to concentrate more on these underrepresented classes. Additionally, significant data augmentation was introduced to enhance the ability to learn features while also guarding against overfitting.
2. The proposed CNN architecture integrates dual track architecture that uses Residual Inception Positional Encoding Attention Network (RIPEANet) with EfficientNetB4. The model leverages both the attention blocks of RIPEANET and pre-trained features to improve classification performance.
3. The integration of Coordinate Attention in the proposed model enhances embedding positional information and cross channel information. This enables the proposed network to focus on important regions with minimal computational cost and improving the accuracy of cassava disease detection. Furthermore, the addition of residual blocks facilitates deeper network training by mitigating the vanishing gradient problem.

## Proposed system

**Fig. 1:**
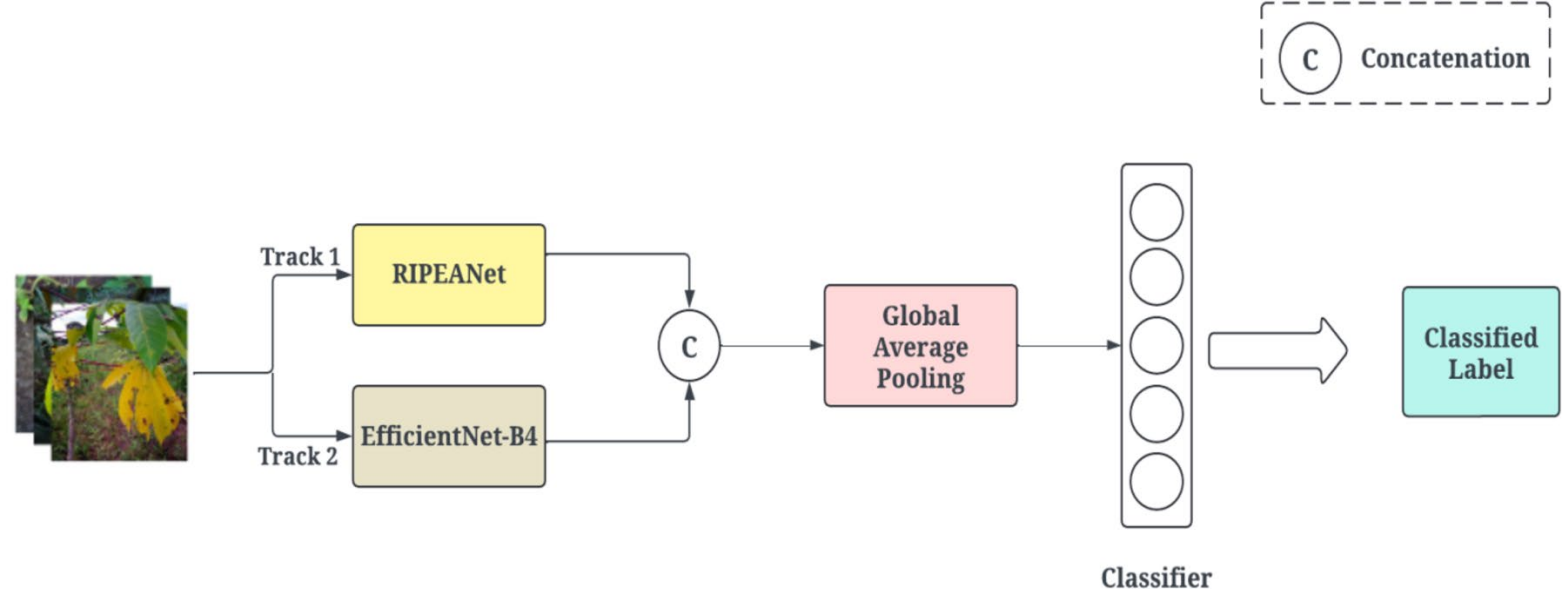
An overview of the hybrid feature aggregation network.

**Fig. 2:**
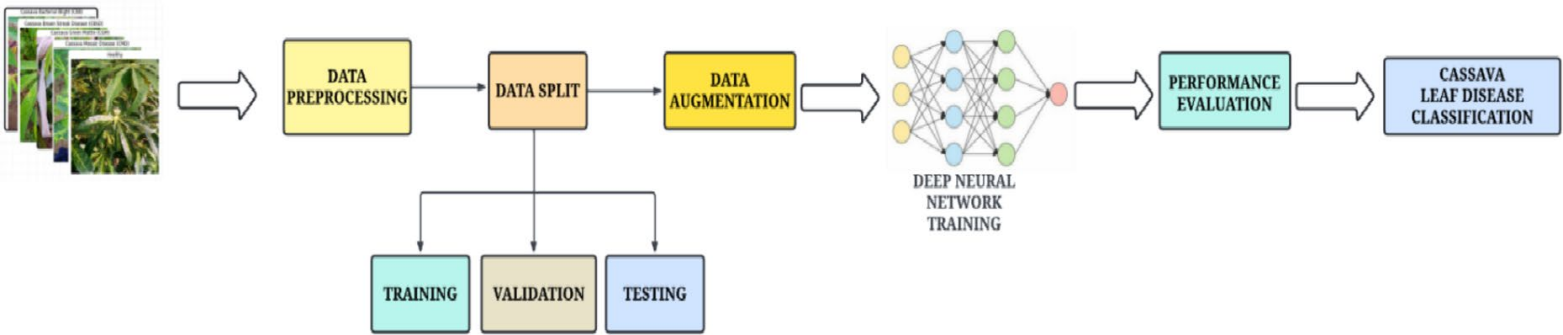
Workflow of the proposed network.

The proposed architecture is a hybrid feature aggregation network that comprises the RIPEANet and EfficientNetB4, as illustrated in Fig. 1. The EfficientNetB4 optimally balances model complexity with computational resources achieving state-of-the-art results. The Coordinate and Mixed attention blocks in RIPEANet ensures both inter-channel and inter-spatial features are focused, thereby providing a feature set that captures various aspects of the input data. Residual links present in the system facilitate the integration of features across multiple scales, enhancing the learning capabilities of the network. Therefore, both the blocks support each other to identify salient regions in the image, leading to more effective classification. The overall workflow of the proposed system is provided in Fig. 2.

### RIPEANet

The first layer of RIPEANet is a convolutional layer with a 7 × 7 kernel, which extracts low-level features such as edges and textures. The use of a large kernel size in this initial layer allows for the capture of extensive spatial information, providing a better framework for subsequent feature extraction. This is followed by a convolution block comprising a 5 × 5 kernel convolution layer, batch normalization, the LeakyReLU activation function, and a max-pooling layer. This block is designed to filter and refine the low-level features while maintaining efficient computation. The RIPEA module plays a crucial role in extracting high-dimensional feature maps. It is applied three times in the architecture to progressively refine and enhance feature extraction at different levels of abstraction. The first RIPEA module focuses on capturing low-level features like edges, textures, and simple patterns. The second module extracts mid-level features, such as more complex shapes and localized disease patterns and the third module focuses on high-level features, integrating global and context-aware information. This module incorporates an Inception-Resnet block that utilizes varying kernel sizes — 1 × 1, 3 × 3, and 5 × 5—providing multi-scale feature extraction with a reduced computational burden.

**Fig. 3:**
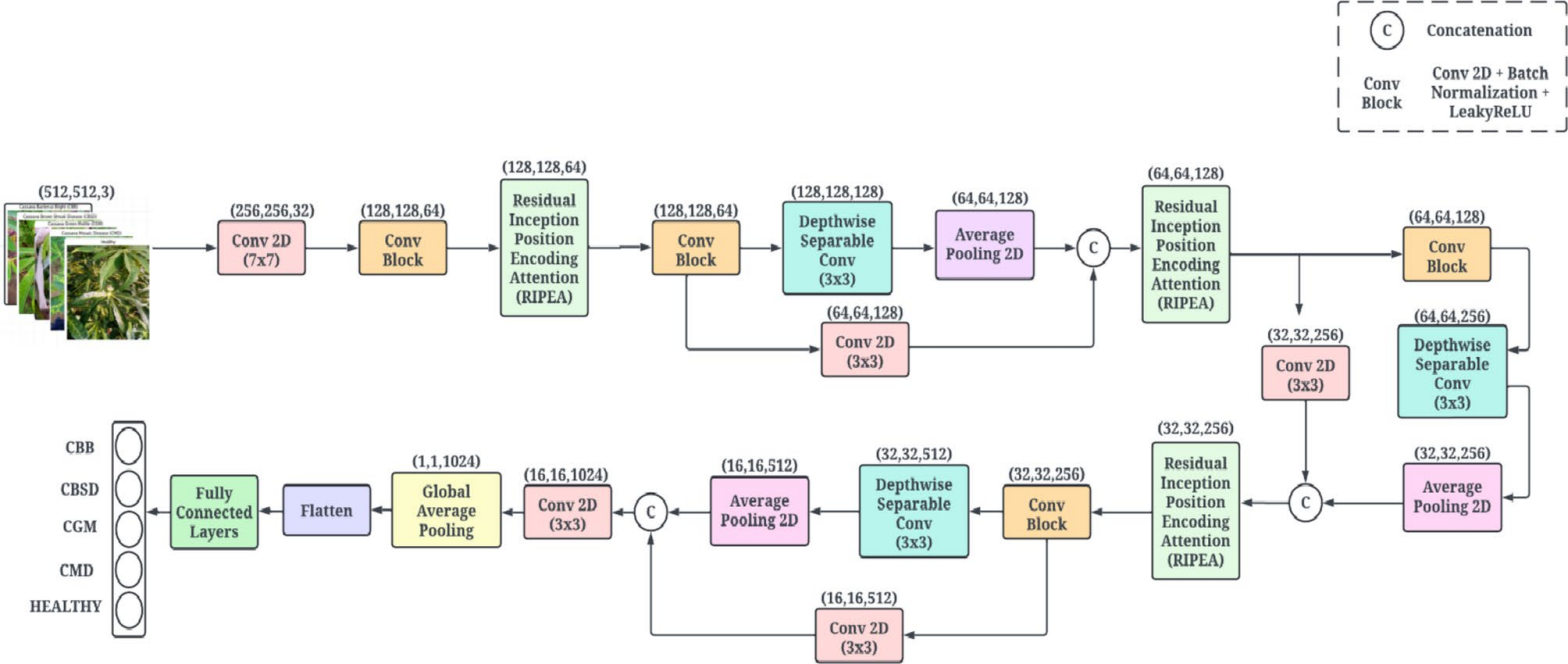
Schematic of the Residual-Inception Positional Encoding Attention Network (RIPEANet).

**Fig. 4:**
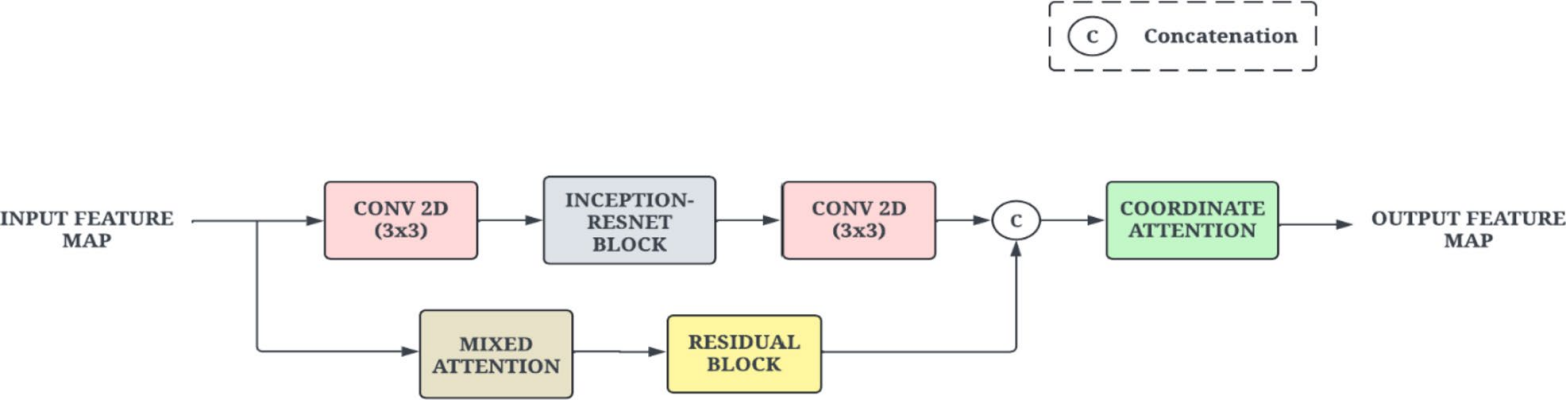
Structure of the RIPEA Module.

In addition to the Inception module, RIPEANet integrates attention mechanisms that focus on both local and global dependencies within the images. These attention modules improve the ability of the network to concentrate on relevant regions of the input images, thereby enhancing feature representation and improving classification performance. The attention mechanisms are particularly beneficial in handling the variations in appearance across different classes of cassava leaf diseases. The inclusion of residual connections ensures that the model retains detailed information from previous layers, preserving essential features of the leaf surfaces. To further optimize the network’s performance, Depth-wise convolutions are employed after each RIPEA module. By independently processing spatial and channel-wise information the model is better able to capture fine-grained textures on leaf surfaces. Additionally, a second path comprising convolutional layers is concatenated with the main track to ensure the network retains important feature information throughout the layers. Global Average Pooling (GAP) is used near the end of the network before the fully connected layers. This preserves spatial information and reduces the likelihood of overfitting, making it effective in the final stages of feature aggregation. The architecture of the proposed RIPEANet is presented in Figs. 3 and 4.

#### Inception-ResNet block

**Fig. 5:**
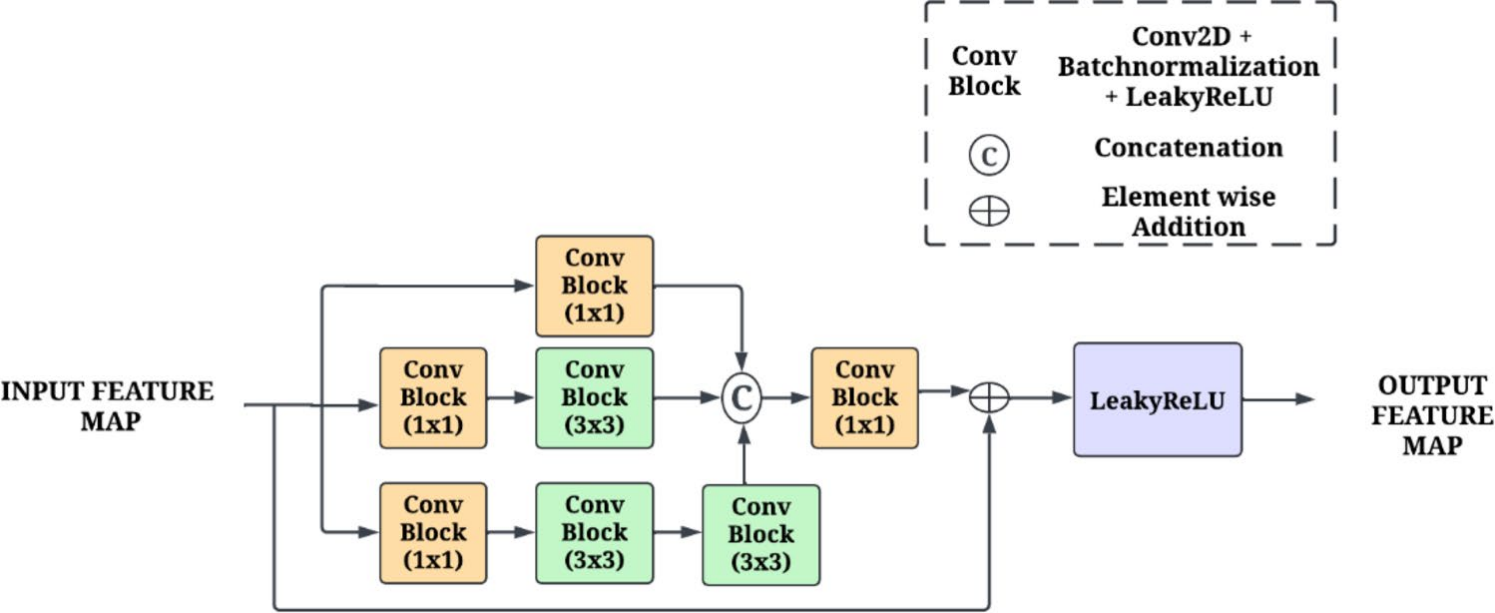
Illustration of the Inception-Resnet Block.

CNNs typically necessitate the use of multiple layers to ensure effective feature extraction. In contrast, models designed to minimize computational costs may compromise their capacity to deliver satisfactory results. The Inception-ResNet block has been used in the RIPEANET architecture to address these challenges^30^. By utilizing parallel convolution with varying kernel sizes that extracts features at multiple scales from input and then concatenates them along channel dimension. Thus, Inception-ResNet lowers computational expenses while producing a high-dimensional feature map. The architecture of this block is given in Fig. 5.

#### Coordinate attention

**Fig. 6:**
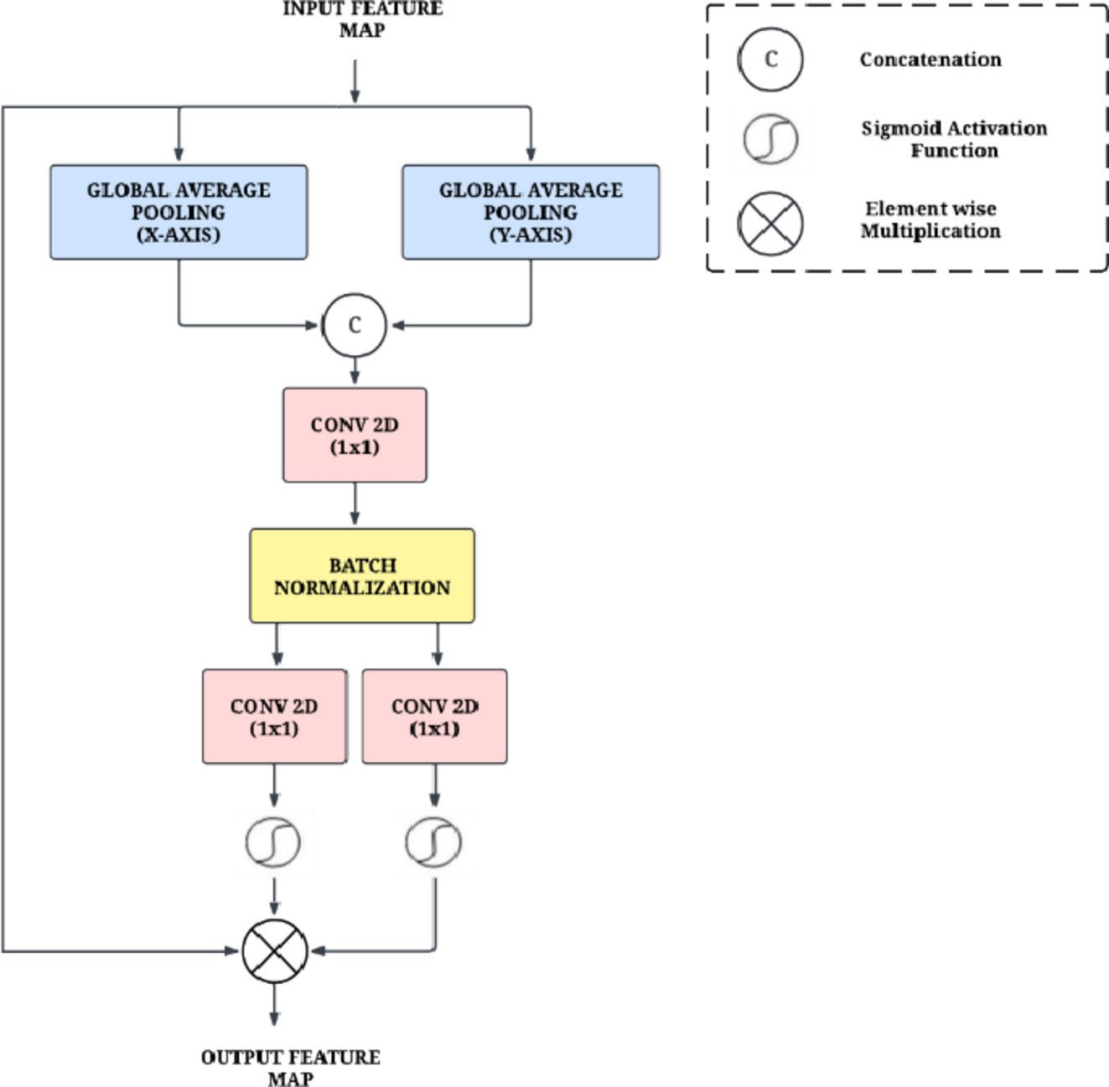
Architecture of the Coordinate Attention.

Coordinate Attention is used in CNNs that selectively pay attention to spatial features in an image^31^. It achieves this by applying GAP along the coordinate axes (x-axis and y-axis) to compute the variance and average of the feature maps for each. Then it combines the mean values to compute attention weights for spatial position by passing through a feedforward network. These attention weights are used to scale the original feature maps, emphasizing important locations and reducing emphasis on the less important ones. Coordinate Attention effectively enhances CNN performance in tasks such as semantic segmentation, object detection, and image classification. Furthermore, this module requires fewer parameters compared to other attention mechanisms, leading to improved computational efficiency and easier integration into CNN architectures. The architecture is presented in Fig. 6.

#### Mixed attention

Mixed Attention is a mixture of self-attention and span-based dynamic convolution^32^. Self-attention (SA) allows the model to focus on different parts of the input sequence by weighing the importance of each element, thus capturing long-range dependencies. On the other hand, Span-based Dynamic Convolution (SDConv) adjusts convolutional filters based on the input, allowing the model to capture local features effectively. The self-attention and span-based dynamic convolution share the same query and utilize different keys to generate their respective attention maps and convolution kernels, thus capturing critical global and local dependencies with reduced redundancy. We formulate the mixed attention given by Eqs. (1), (2), and (3):

1 $$\:SA(Q,K,V)=softmax\left(\frac{{Q}^{T}K}{\sqrt{d}}\right)$$

**Fig. 7:**
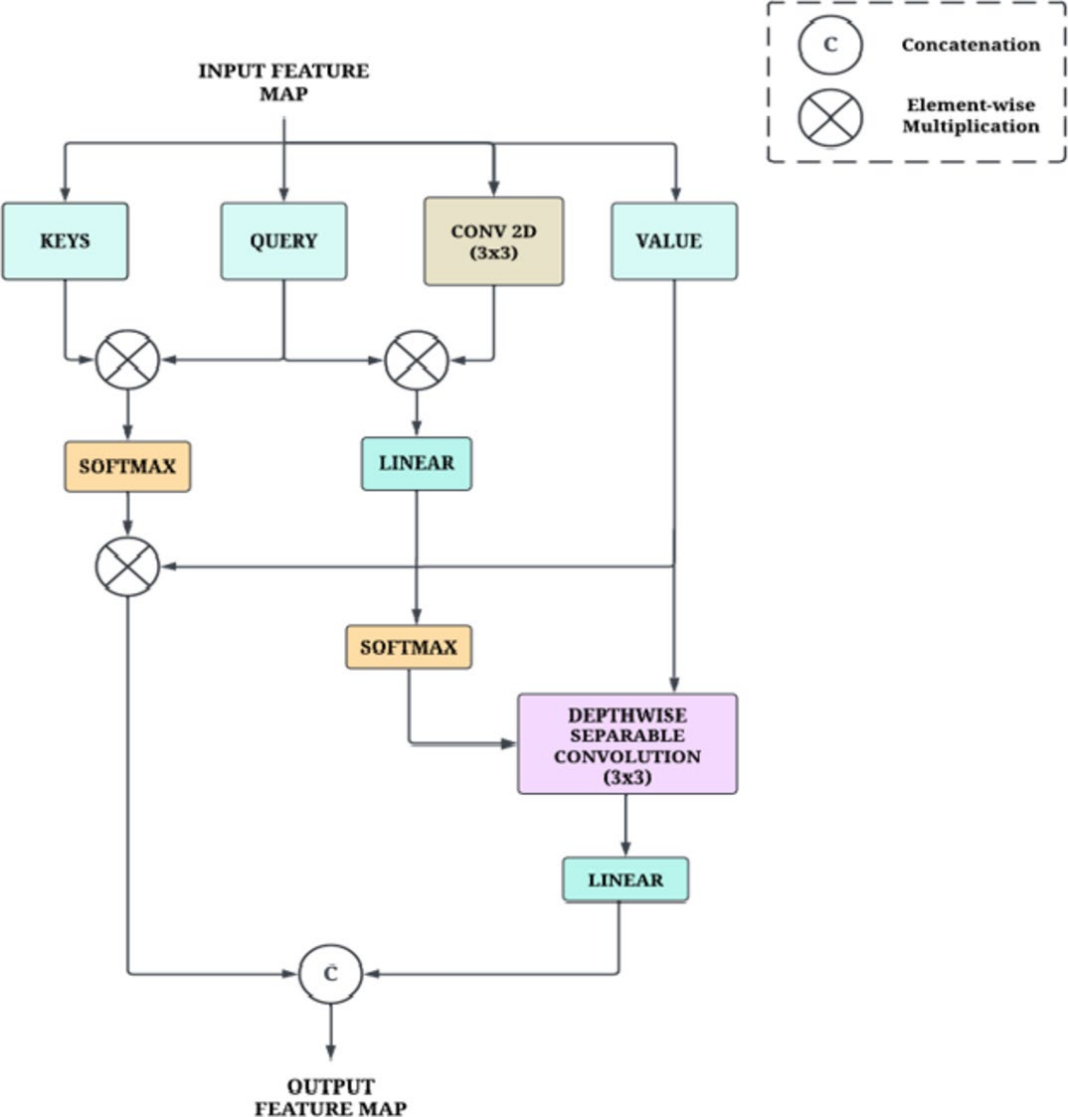
Architecture of Mixed Attention.

Here ‘Q’ denotes query, ‘K’ is keys, ‘V’ is value, and ‘H’ is the hyperparameter that determines the number of attention heads. For H self-attention heads, the query, value, and key embeddings are divided into equal-dimensional segments, where each segment has a dimensionality of dk = d/H.

2 $$\:SDConv\left(Q,{K}_{s},V;{W}_{f},i\right)=DeptConv\left(V,soft{max}\left({W}_{f},\left(Q\odot\:{K}_{s}\right)\right),i\right)$$

3 $$\:MixedAttn\left(K,Q,{K}_{s},V;{W}_{f}\right)=Cat\left(SelfAttn\left(Q,K,V\right),SDConv\left(Q,{K}_{s},V;{W}_{f}\right)\right)$$

where Cat (,) is the concatenation operation, ‘⊙’ represents pointwise multiplication, and ‘∙’ represents multiplication operation. ‘X’ is the input tensor, ‘d’ is the hidden dimension, ‘i’ denotes position, ‘Ks’ is the span-aware key, DeptConv represents lightweight depth-wise separable convolution, and ‘W’ denotes the convolution kernel. Figure 7 provides an illustration of the mixed attention architecture.

### Feature concatenation and classification layers

The feature maps from RIPEAnet and EfficientNet tracks are aggregated for further processing. This feature fusion leverages the strengths of both tracks. Track 1 captures fine-grained spatial details using RIPEANet, while Track 2 focuses on more abstract and high-level representations using EfficientNetB4. The fusion strategy is designed to enhance feature diversity while ensuring integration of relevant features. RIPEANet extracts hierarchical features through Inception-ResNet blocks and attention mechanisms, preserving local textures and disease-specific patterns. EfficientNetB4 acts as a global feature extractor, leveraging Mobile Inverted Bottleneck Convolutions (MBConv) and Squeeze-and-Excitation modules to enhance feature abstraction. To ensure feature compatibility before fusion, a 1 × 1 convolution layer is applied to standardize dimensional differences between feature maps. The processed feature representations from both tracks are concatenated along the channel axis, enabling integration of low-level textures and high-level contextual information. This method ensures that disease-relevant local features extracted by RIPEANet complement global leaf structure information derived from EfficientNetB4. A GAP layer is applied to the concatenated feature maps, reducing spatial dimensions while retaining essential discriminative features. The pooled feature representation is flattened and passed through fully connected layers, ensuring optimal classification into cassava disease categories. The final classification is performed using a softmax layer, mapping the extracted representations into one of the predefined disease classes.

## Results and discussion

This section outlines the dataset details, preprocessing techniques, and highlights the data augmentation methods employed in this work. It further presents the findings of the study, including the environmental setup and results from the ablation studies. The following sections include a visual representation of the features utilizing Grad-CAM, accompanied by a comparative analysis with state-of-the-art networks.

### Dataset description

**Fig. 8:**
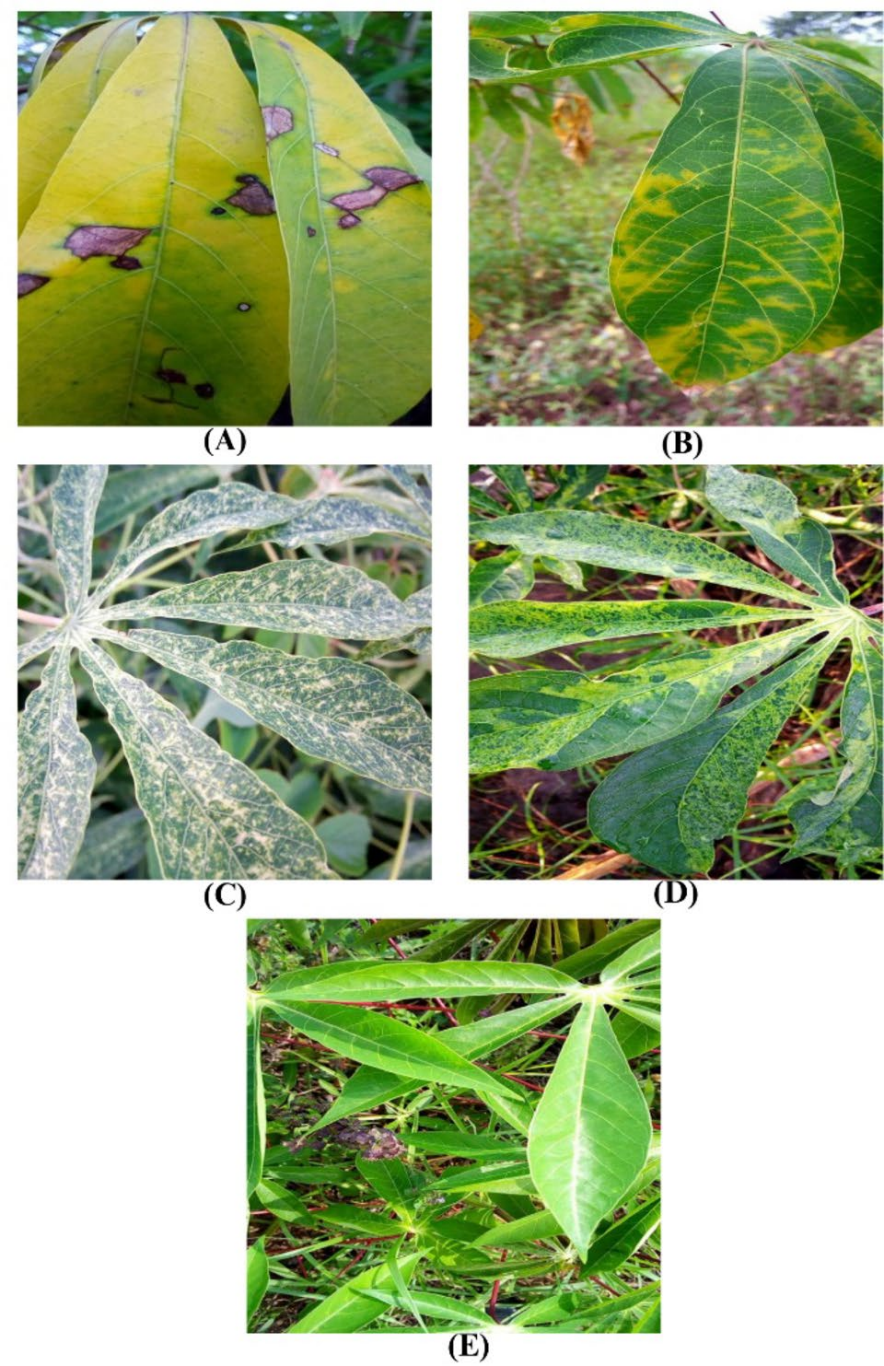
Sample leaf images from the Cassava dataset: (A) CBB (B) CBSD (C) CGM (D) CMD (E) Healthy.

**Fig. 9:**
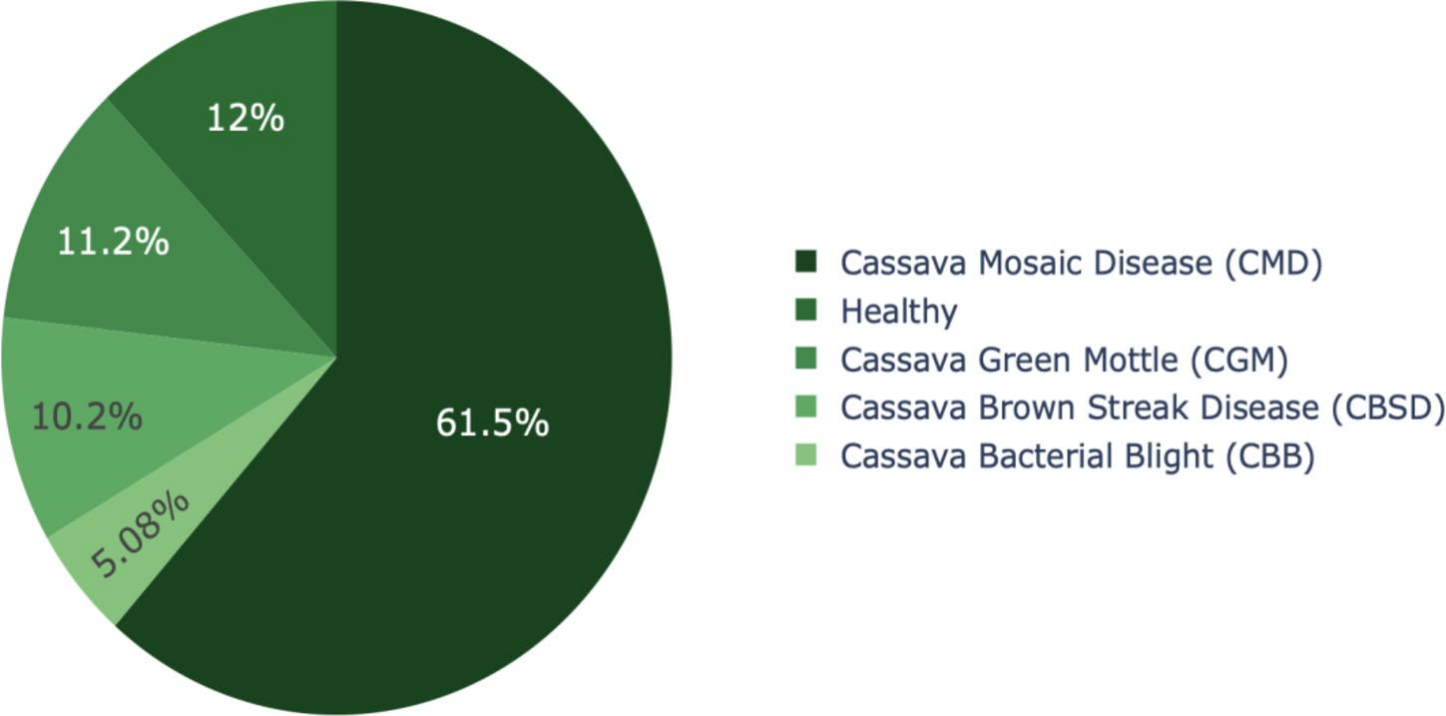
Class distribution of image samples in the dataset.

The dataset used in this research work was obtained from the Makerere Artificial Intelligence Lab^33^. The five primary categories comprise CMD, CBB, CBSD, CGM, and Healthy Leaf, as illustrated in Fig. 8. There is a severe class imbalance due to more than half of the image samples belonging to the CMD class. Figure 9; Table 1 provides the number of samples per class of the dataset.

**Table 1:** Cassava leaf disease dataset details.

Sample Class	Number of Image Samples
Cassava Bacterial Blight (CBB)	1087
Cassava Brown Streak Disease (CBSD)	2189
Cassava Green Mottle (CGM)	2386
Cassava Mosaic Disease (CMD)	13,158
Healthy	2577
Total	21,397

### Data augmentation

The preprocessing and augmentation techniques employed for the dataset are important for enhancing the performance of the model. The dataset is divided into 60% for training, 20% for validation, and 20% for testing. Considering the substantial class imbalance, the model can have biased performance with over 50% of the images belonging to the CMD class. To mitigate this, data augmentation techniques were employed utilizing the Keras and Pillow libraries: (1) Random horizontal and vertical flipping to provide the model with a variety of image orientations; (2) Random transpositions of images to further diversify the training samples; and (3) Adjusting brightness within the range of 0.7 to 1.3 to account for varying lighting conditions. These augmentations not only helped balance the dataset but also enhanced the generalization of the model by exposing it to a broader spectrum of possible real-world scenarios.

Horizontal and vertical flips were applied to counteract the directional bias in leaf orientation, ensuring that the model learns disease patterns independent of leaf alignment. Rotation transformations were introduced to simulate the natural variability in leaf positioning due to differences in manual or automated image capture methods. Width and height shifts were incorporated to account for spatial displacements, ensuring that disease features are learned irrespective of precise leaf positioning within the frame. Zoom transformations were applied to address differences in image capture distances, making the model invariant to varying scales of leaf structures. Brightness and contrast adjustments were utilized to simulate varying lighting conditions encountered in agricultural environments, preventing over-reliance on uniform illumination for classification. These augmentations collectively enhance the model’s ability to accurately identify cassava leaf diseases across diverse real-world scenarios, mitigating biases associated with dataset collection conditions.

### Environmental setup

The proposed architecture was implemented using Keras, an open-source framework based on the Python programming language. All training and testing were performed using a Kaggle notebook with an Nvidia P100 GPU. To find optimal parameters, Adaptive Moment Estimation with weight decay (AdamW) with initial learning rate of 0.00001 was used. To prevent the model from getting stuck at local minima and improve convergence, a learning rate scheduler called cosine decay with warm restarts was utilized. The cosine decay function progressively reduces the learning rate according to a cosine function, allowing for gradual and fine-tuned weight updates. The warm restarts mechanism, determined by parameters such as ‘first_decay_steps’ (the number of steps before the first restart), ‘t_mul’ (the factor by which the number of iterations increases after each restart), and ‘m_mul’ (the factor by which the learning rate decreases after each restart), permits periodic resets of the learning rate, effectively helping the model escape local minima and enhancing convergence during training.

### Ablation studies

Ablation experiments were conducted to validate the performance of different components in the proposed architecture. This helps to systematically analyze the contribution of each component by removing or modifying specific elements and observing the impact on overall performance of the proposed model.

#### Analysis of EfficientNetB4 network

**Fig. 10:**
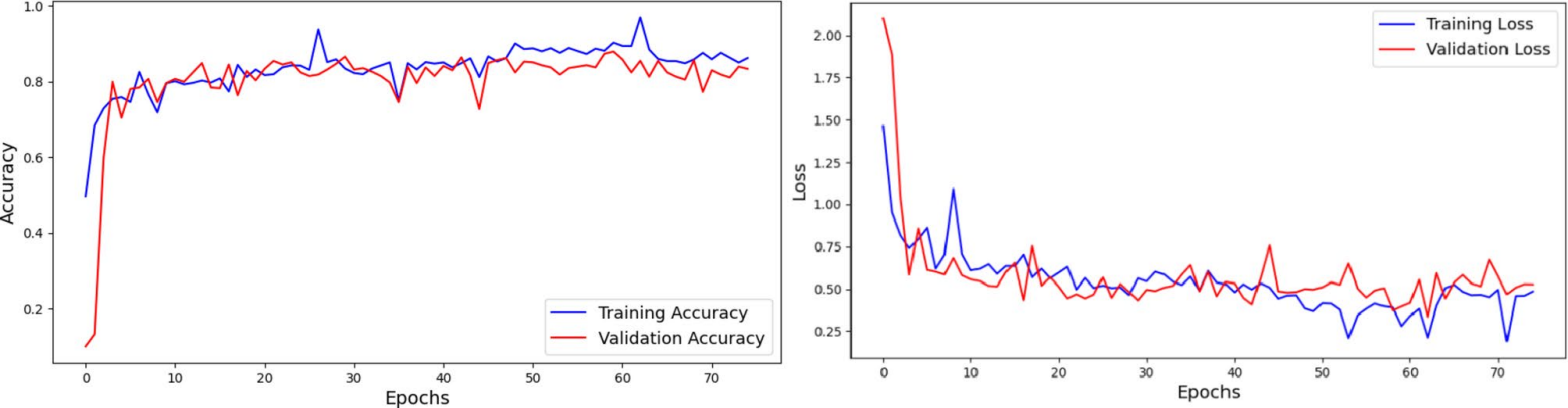
Analysis of EfficientNetB4 Architecture using Accuracy and Loss Plots.

The performance of the EfficientNetB4 model is discussed in this subsection. The compound scaling approach of the EfficientNetB4 adjusts the depth, width, and resolution of the network in a balanced way. Unlike traditional methods that only scale one of these dimensions, EfficientNetB4 optimizes all three, leading to a more efficient model without compromising accuracy. The compound scaling approach also ensures that the model can generalize well to different image resolutions. The architecture is optimized through Mobile Inverted Bottleneck Convolution (MBConv) blocks combined with squeeze-and-excitation networks, enhancing its feature extraction capabilities. The structure of the model, which includes a series of blocks with varying resolutions, allows it to focus on different levels of detail within the leaf images. This is important for accurately distinguishing between subtle differences in leaf texture and color. The model underwent training for 75 epochs. The observation graphs are presented in Fig. 10.

#### Analysis of ripeanet without coordinate attention

**Fig. 11:**
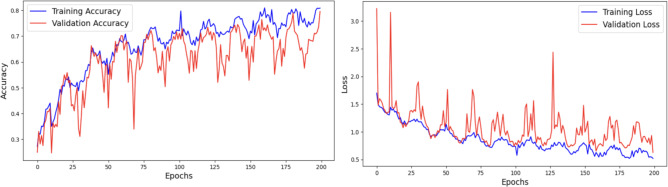
Analysis of RIPEANet without Coordinate Attention using Accuracy and Loss Plots.

This experiment examines the outcomes obtained by combining the features of the RIPEANet, without incorporating the Coordinate Attention. The model was trained with the AdamW optimizer and Categorical Cross-entropy loss function. The Coordinate Attention enhances the performance of RIPEANet by integrating positional information into channel attention. In the absence of this block, the RIPEANet model exhibits increased variability between training and validation loss during the 200-epoch training period. An overall accuracy of 78.79% was achieved in the test set. Figure 11 presents the accuracy and loss graphs respectively.

#### Analysis of ripeanet with coordinate attention

**Fig. 12:**
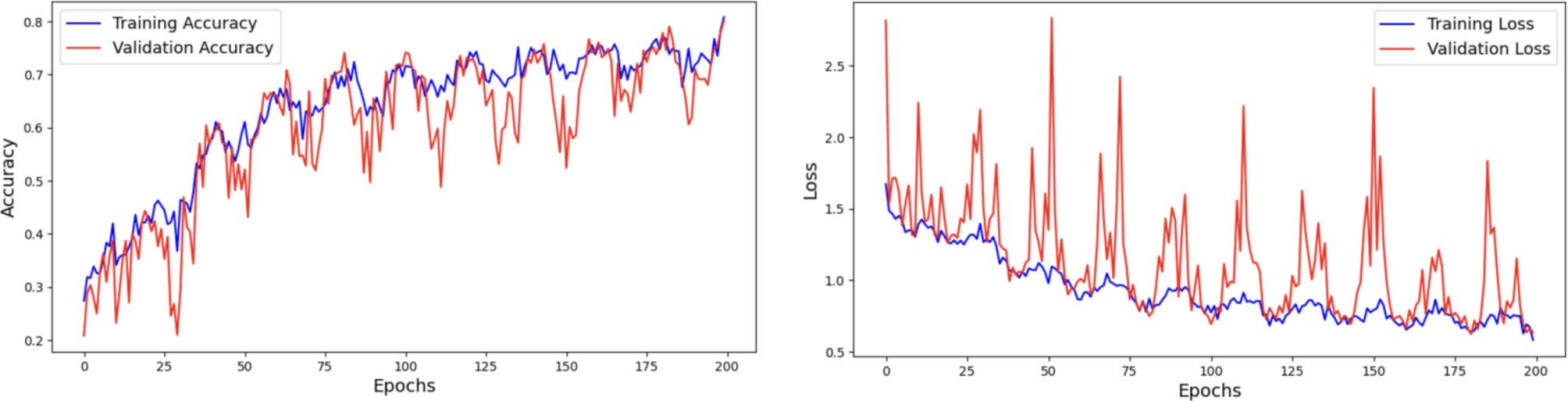
Analysis of RIPEANet with Coordinate Attention using Accuracy and Loss Plots.

Through the integration of Mixed Attention block and Coordinate attention block, RIPEANet significantly improves feature extraction by improving the ability of the network to identify and highlight relevant features. It specifically improves the ability of the model to capture and utilize spatial relationships within the feature maps by focusing on spatial details along both axes. This allows the network to maintain a larger receptive field and more effectively model cross-channel relationships. RIPEANet achieves more precise localization of relevant features, leading to better overall accuracy. Figure 12. illustrates the accuracy and loss graphs respectively with a final test accuracy of 81.46% achieved after 200 epochs.

#### Analysis of the proposed network

The feature maps generated by RIPEANet are concatenated with those from EfficientNetB4, creating a representation that includes both the fine-grained, localized features from RIPEANet and the broader, high-level features from EfficientNetB4. This combination enables the model to capture a broad range of relevant information from local texture details to global structural patterns and improves the overall classification accuracy. This dual-track architecture reduces the susceptibility of the model to overfitting by fusing feature maps and incorporating diverse feature representations. This approach achieves 93.06% accuracy on the test set. The advantages of this hybrid feature aggregation approach are evident in its ability to balance detailed feature extraction with efficient computation, making it suitable for applications where both better accuracy and computational efficiency are required. The proposed dual-track network exhibits balanced precision, recall, and F1-scores across all classes. The model effectively distinguishes between cassava leaf diseases and the healthy class, as demonstrated by the consistently high recall and F1-score across all classes. The integration of multi-scale local feature representations for RIPEANet and global contextual representations from EfficientNetB4 strengthens the model’s ability to capture fine-grained disease patterns. This enhances the capacity to recognize broader structural information.

**Fig. 13:**
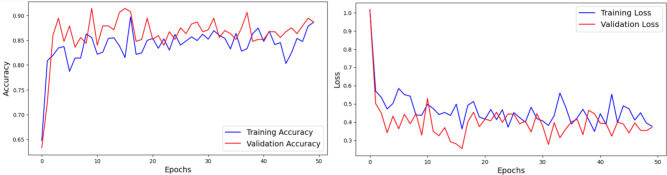
Analysis of proposed network using non-augmented dataset using accuracy and loss plots.

**Fig. 14:**
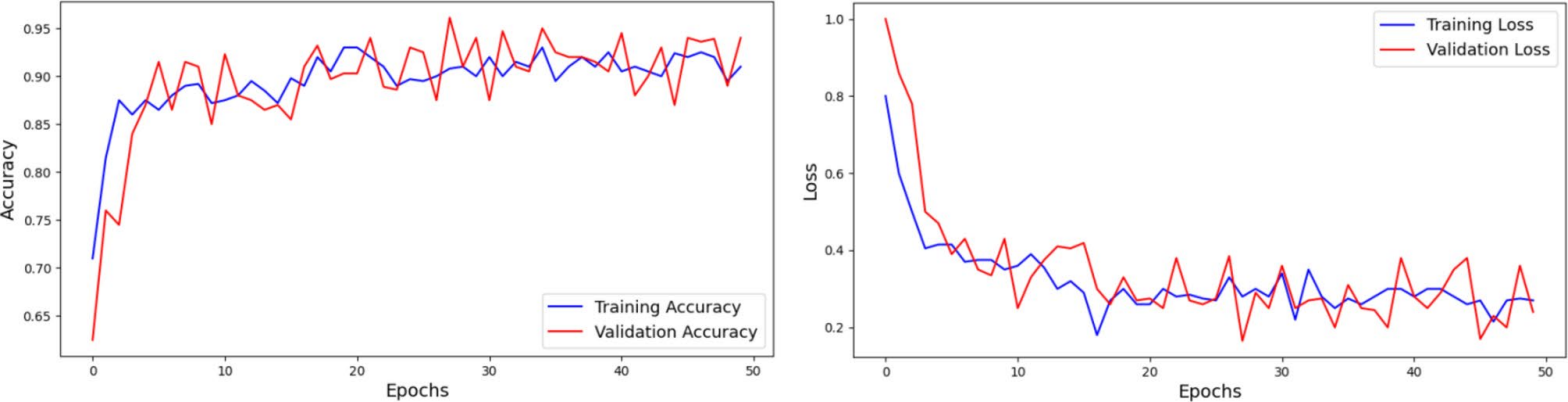
Analysis of the proposed hybrid feature aggregation network using accuracy and loss Plots.

**Fig. 15:**
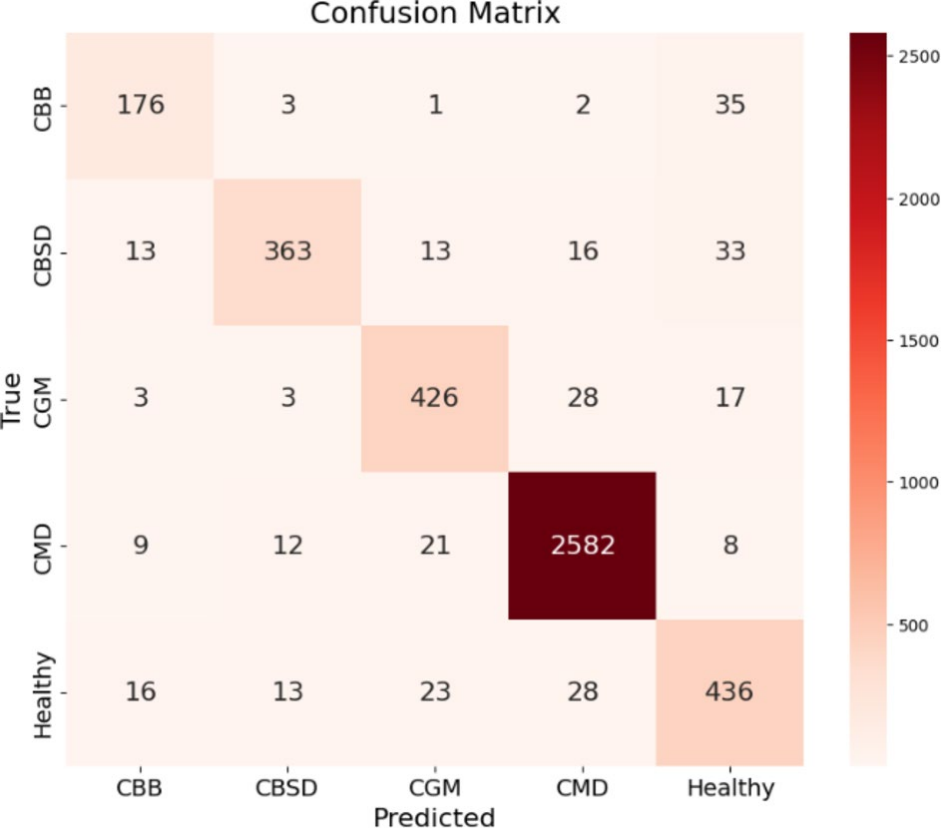
Confusion Matrix Depicting Predictions Across CBB, CBSD, CGM, CMD, and Healthy Categories.

The proposed network was also trained on the non-augmented dataset for more detailed analysis. The results demonstrated improved evaluation metrics when training with augmented data. Classification results of underperforming classes such as CBB and Healthy classes were improved after augmentation. The graphs of the study are illustrated in Fig. 13. The experimental results and confusion matrix of augmented training results are illustrated in Figs. 14 and 15 respectively. Table 2 presents the comparison of class-wise metrics between augmented and non-augmented dataset.

**Table 2:** Class-wise metrics for the proposed network.

Classes	Precision		Recall		F1-Score		Support
	Non-augmented	Augmented	Non-augmented	Augmented	Non-augmented	Augmented	
CBB	0.631	0.811	0.591	0.811	0.609	0.811	217
CBSD	0.767	0.921	0.783	0.828	0.775	0.872	438
CGM	0.791	0.880	0.719	0.893	0.751	0.886	477
CMD	0.956	0.972	0.946	0.981	0.950	0.976	2632
Healthy	0.664	0.824	0.759	0.845	0.707	0.833	516

Table 3 summarizes the results from different experiments to understand how each component of the model contributes to its overall performance. EfficientNetB4 model as a standalone model achieved an accuracy of 88.09%. Without Coordinate Attention, RIPEANet showed an accuracy of 78.79%. However, when Coordinate Attention was added, RIPEANet’s performance improved to 81.46%, an increase of 2.67%. The best results were seen with the proposed hybrid feature aggregation network, which combines both tracks and achieved an accuracy of 93.06%, along with higher precision and recall values.

**Table 3:** Analysis of the ablation experiments made.

S. No	Experiment	Number of Parameters (M)	Accuracy (in %)	Precision (in %)	Recall (in %)
1	EfficientNetB4 Track	19.5	88.09	88.98	86.60
2	RIPEANet without Coordinate Attention	22.1	78.79	76.20	76.42
3	RIPEANet with Coordinate Attention	29.9	81.46	83.10	81.46
4	Proposed Network (Non-augmented training)	49	86.33	86.58	86.33
5	**Proposed Hybrid Feature Aggregation Network**	**49**	**93.06**	**88.18**	**87.18**

## Discussion

In this section, a detailed visual analysis of the features learned by the proposed system is presented. Additionally, the results of the proposed system are compared with existing studies and state-of-the-art CNNs.

### Visual representation of features using Grad-CAM

Gradient-weighted Class Activation Mapping (Grad-CAM) was used to visualize diseased regions of cassava leaves, as shown in Table 4. This illustrates the effectiveness of the proposed network in accurately identifying and localizing diseased areas. Grad-CAM provides visual explanations by highlighting the regions in the input images that contribute most significantly to the network’s predictions. In the context of the proposed system, Grad-CAM was applied to the second to last convolutional layer, which preserves crucial spatial information about the input images. By leveraging the gradients from this layer, the network generates heat maps that delineate the diseased regions of the leaves. This is especially significant for cassava leaf disease classification, where symptoms often appear in specific patterns on the leaf surface. The concentrated heatmaps observed in the Grad-CAM visualizations indicate that the proposed network is effectively focusing on the critical areas that are most indicative of the presence of disease. This not only validates the network’s ability to learn discriminative features but also provides interpretability, to understand how the model arrives at its decisions.

**Table 4:**
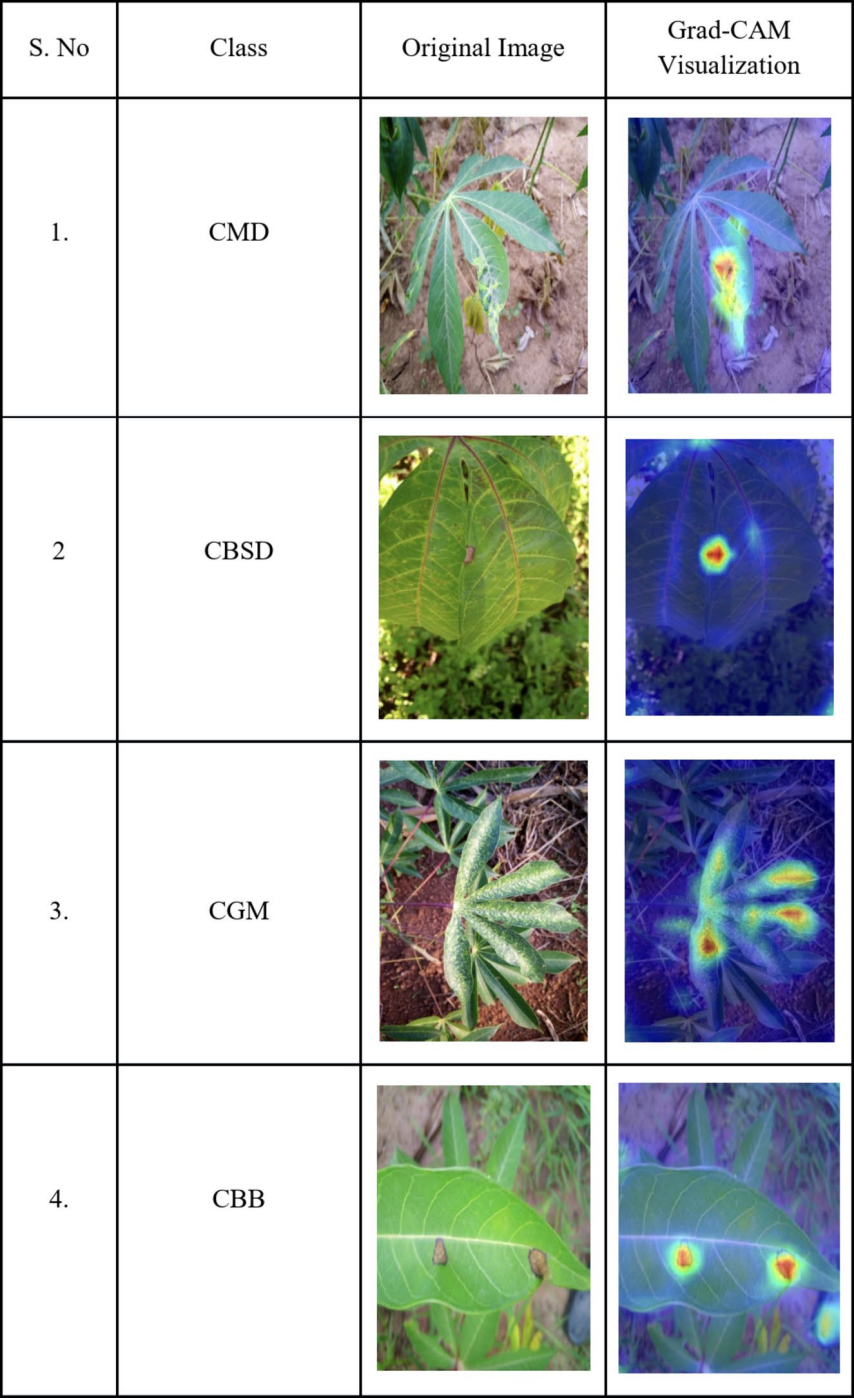
Visualizing diseased regions of cassava leaves using Grad-CAM.

### Performance analysis with state-of-the-art networks

The comparison of various state-of-the-art CNN architectures with the proposed dual-track network on the cassava leaf disease dataset is presented in Table 5. VGG16, exhibits the lesser performance, with an accuracy of 77.56% and F1-score, indicating that increasing model complexity does not necessarily lead to better results. AlexNet underperforms with 77.97% accuracy and lower metrics, highlighting its limitation in capturing the complex patterns needed for accurate cassava leaf disease classification. Xception, EfficientNet and ResNet50, despite being parameter-efficient, achieved accuracy of 77.0%, 84.0% and 84.25%. DenseNet provides an accuracy of 79.25% and other metrics, further adapting generalized architectures to this cassava leaf disease detection. The proposed hybrid aggregation network achieves 93.06% accuracy and improved macro-metrics by integrating feature fusion techniques and effectively addressing class imbalance, making it suitable for cassava leaf disease classification.

The proposed network contains 49 million trainable parameters making it significantly smaller than models like VGG16, and AlexNet. The study focused on achieving high accuracy with real-field images while carefully considering the trade-off between model complexity and computation efficiency. Understanding the trade-offs between model complexity, inference speed, and accuracy is essential when selecting an appropriate architecture for real-world applications. While a larger model may offer superior predictive performance, it is crucial to balance computational efficiency, especially for deployment in resource-constrained environments.

**Table 5:** Comparison of the performance of state-of-the-art CNN architectures.

Architecture	Number of Parameters (M)	Accuracy (in %)	Precision (in %)	Recall (in %)	F1-score (in %)	Inference Time (per sample)
Xception	22.8	77.0	62.0	54.0	57.72	0.237
VGG16	138	77.56	61.89	59.25	60.56	0.155
AlexNet	57	77.97	61.20	55.69	58.30	0.612
DenseNet	7.3	79.25	67.34	55.45	60.82	0.625
Resnet50	24.11	84.25	74.0	69.30.	71.61	0.213
EfficientNet	4.37	88.09	88.98	88.60	88.79	0.378
**Proposed Network**	**49**	**93.06**	**88.18**	**87.18**	**87.62**	**0.088**

### Comparison with existing studies

The comparative analysis of the proposed network with the existing research studies utilizing cassava leaf disease dataset is presented in Table 6. The results indicate that the proposed network achieves an accuracy of 93.06%, surpassing previous studies. Use of Simple Framework for Contrastive Learning of Visual Representations (SimCLR) with hybrid loss functions shows an accuracy of 91.59%, highlighting the potential of self-supervised learning approaches. ResNest-59 and Lightweight modified attention custom CNN achieve accuracies of 89.70% and 75.00%, respectively. This reinforces the notion that a custom architecture tailored to the dataset, as seen in the proposed network, offers significant performance improvements.

**Table 6:** Analysis of the proposed work in comparison to current studies that have utilized the Makerere university AI lab’s cassava leaf disease dataset.

S. No	Source	Model	Accuracy (in %)
1	Tewari et al.^34^	Lightweight Modified Attention-based Network	75.00
2	Methil et al.^15^	Transfer Learning EfficientNet-B4	85.64
3	Singh et al.^35^	InceptionResNetV2	87.86
4	Maryum et al.^29^	Transfer Learning EfficientNet-B4	89.09
5	Chen et al.^36^	Transfer Learning ResNest-59	89.70
6	Zhang et al.^37^	SimCLR	91.59
7	Vijayalata et al.^38^	Transfer Learning EfficientNet-B0	92.60
**8**	**Proposed Network**	**Hybrid Feature Aggregation Network**	**93.06**

### Limitations and future work

The following are some of the limitations of the proposed system. It also proposes the scope for future research.

1. Although the model achieves high classification accuracy, its adaptability to varied agricultural environments remains untested. Real-world cassava fields present challenges such as overlapping foliage, mixed infections, and fluctuating environmental conditions that may affect disease presentation. While data augmentation introduces variability, it cannot fully capture these complexities. Evaluating the model across geographically diverse datasets with different environmental factors would improve its generalizability and robustness in practical deployment.
2. The study also does not account for the temporal progression of cassava diseases, as classification is based on static images. Symptoms evolve over time, making early-stage infections harder to distinguish from transient environmental effects. Incorporating sequential image data could enhance disease tracking and improve early detection, with future work exploring temporal modeling approaches like recurrent or transformer-based networks.
3. Additionally, the computational demands of the hybrid feature aggregation network may limit its feasibility in real-time or mobile applications. The integration of multiple attention mechanisms improves accuracy but increases inference costs, making deployment in resource-constrained settings challenging. Optimizations such as pruning, quantization, or knowledge distillation could reduce complexity while preserving performance, enabling broader accessibility in practical agricultural monitoring systems.

## Conclusion

Cassava is a vital crop for millions and faces significant threats from various diseases due to diverse environmental conditions. The manual observation methods employed in current disease identification approaches are labor-intensive, could lead to delayed responses, and cause yield losses. The main goal of the research is to accurately classify cassava leaf disease by utilizing a DL architecture. The proposed dual track model integrates RIPEA network and EfficientNetB4 for effective classification. The model captures multi-scale features through the Inception-ResNet block and emphasizes important spatial and channel features through mixed attention mechanism. Additionally, the model utilizes Coordinate Attention to integrate positional information into channel attention, thereby enhancing feature extraction and increasing the accuracy of the DL model. The proposed model also applies class weights to address class imbalance, which enhances feature learning through extensive data augmentation. The use of residual blocks used in the proposed network assists in alleviating the vanishing gradient issue, enabling the training of deeper networks. The fine-tuned features from RIPEA network and EfficientNetB4 are integrated to achieve accurate classification of cassava leaf disease. This method attained a classification accuracy of 93.06%, highlighting its ability to address class imbalance and improve overall model performance.

## Data Availability

The datasets generated and/or analyzed during the current study are available in Kaggle. link: https://kaggle.com/competitions/cassava-leaf-disease-classification.
